# Acute intermittent hypoxia: Enhancing motoneuronal output or not?

**DOI:** 10.1113/EP091985

**Published:** 2024-06-25

**Authors:** Simon C. Gandevia, Jane E. Butler

**Affiliations:** ^1^ Neuroscience Research Australia and University of New South Wales Randwick Australia

**Keywords:** acute intermittent hypoxia, TMS

## 1 INTRODUCTION

Connections link a sequence of three related research papers. The central article which links the other two papers has been published in *Experimental Physiology*. In a Connections article, an author (or authors) of the central article outlines its principal novel findings, tracing how they were influenced by the first article and how the central article has contributed to the developments made in the third article. The author(s) may also speculate on the direction of future research in the field. Connections articles aim to set the research in a wide context.

Motoneurone output is crucial for all reflex and voluntary movements. Furthermore, this output is compromised in conditions affecting motoneurones, their local intraspinal circuits and descending corticofugal drives. This justifies the need to understand the physiology of motoneurones and to search for therapeutic strategies to repair and rehabilitate their output when impaired. Of course, outputs from respiratory motoneurones are prioritised in any hierarchy of output because they drive pulmonary ventilation.

Short alternating periods of hypoxia and normoxia (acute intermittent hypoxia, AIH) have been shown in animal studies, first done more than 30 years ago, to enhance the output of respiratory motoneurones, particularly those in the phrenic motor nucleus. The responsible post‐synaptic pathways and neurophysiological mechanisms have been well explored (e.g., Navarrete–Opazo & Mitchell, 2014). This seemingly straightforward way to boost motoneurone output has been taken up and used to enhance (or not) motoneurone output in humans with disorders such as stroke and spinal cord injury.

This Connections piece begins with a description of a test of a major corollary of up‐regulation of the output from human motoneurones by AIH via post‐synaptic mechanisms (in healthy humans without a spinal cord injury). In a simple, double‐blinded, randomised, cross‐over design, Finn et al. (2022) asked whether the Hoffman (H) reflex in the soleus muscle, which is largely monosynaptic, was altered by a period of intermittent hypoxic breathing. The duration and severity of the hypoxia (9% O_2_) was typical of prior studies. The central dogma would say this intervention would enhance the post‐synaptic effect of the primary muscle spindle input on soleus motoneurones. They were studied under controlled conditions with the participants at rest. In this study there was no consistent change in the size of the reflex over the 60 min after the AIH intervention. This was based on an assessment of the H reflex recruitment curve and the maximal H‐reflex. The maximal M‐wave was unchanged.

In addition, Finn et al. (2022) asked whether the motor potential evoked by transcranial magnetic stimulation (MEP) in an intrinsic hand muscle changed after the AIH intervention. Again, the evoked responses include a strong monosynaptic component (corticospinal) and they were measured during relaxation. This was motivated by the prior study of Christiansen et al. (2018) who had also studied this response and showed it increased in all participants. Across the group of participants, Finn et al. (2022) found AIH failed to produce a coherent change in corticospinal evoked responses. However, despite these generally negative findings, Finn et al. (2022) talked up some ‘statistically significant’ amplifications of evoked reflex and corticospinal responses which ‘were inconsistent and only at distinct time intervals’. Of the 120 pre‐planned *post hoc* tests of AIH efficacy, five were statistically significant at the conventional 5% level (Finn et al., 2022; supplementary information S1)—hardly a reason to head to the casino!

So, what were the dramatic findings of Christiansen et al. (2018)? They focused on the corticospinal pathway to a hand muscle—from motor cortex to motoneurone. First, in the hour after the AIH intervention, the effect of motor cortical stimulation, but also importantly subcortical stimulation of corticospinal axons, was facilitated significantly above baseline (by ∼40%, SD ∼30%, and by ∼75–100%, SD ∼75%, respectively). (This was estimated crudely from their Figs 2 and 4 for the 15–75 min interval after the intervention.)

Second, another expression of motoneurone plasticity (spike timing‐dependent plasticity) based on the temporal interaction between a descending (corticospinal) input to the motoneurones and separate (antidromic) activation of the motoneurone showed upregulation (>50%, their Fig. 6) after the AIH intervention. Finally, an indirect, albeit controversial, measure of the ‘intrinsic excitability’ of the motoneurone (Ozyurt et al., 2024), its ability to be activated by an antidromic action potential (the F‐wave) was not changed by the AIH intervention.

The simplest unifying conclusion from Christiansen et al. (2018) is that AIH acts at the level of the corticospinal synapses onto motoneurones. Disappointingly, while this may have prompted the study by Finn et al. (2022) described earlier, any facilitation of corticospinal action on motoneurones seems not to occur uniformly within studies or between them. Radia et al. (2022) also found no influence of AIH on corticospinal output to a hand motoneurone pool. They used a slightly different intermittent protocol, but it still involved 15 min of hypoxia in total. More recently, Mathew et al. (under review) 1found no change in MEPs after single sessions of AIH on corticospinal output to the hand or to the lower limb.

So far, the only published study on changes in a mostly monosynaptic spinal reflex by AIH is that by Finn et al. (2022) with minimal to no effect. However, the failure of AIH to enhance reflex output has recently been confirmed for the stretch reflex of soleus (Tan et al., 2024).

Where does this leave us? Perplexed. But, a study by Welch et al. (2022) adds a further dimension to the physiological processes being explored with AIH. It is the level of CO_2_. In a typical AIH protocol, the mild over‐ventilation, due to an increased tidal volume, reduces end‐tidal CO_2_ in some cases (e.g., Finn et al. 2022) but not others (Welch et al., 2022). How does the CO_2_ level interact with the putative neuroplasticity at the motoneurone pool? Using transcranial magnetic stimulation to elicit MEPs in the diaphragm with indirect chest‐wall recordings, Welch et al. (2022) found that the typical AIH intervention did not alter the size of the MEPs, but if they added 4% CO_2_ to the hypoxic gas (9% O_2_), facilitation of the corticospinal output to phrenic motoneurones appeared. It is not known whether this would occur for limb motoneurones.

It is disappointing that a proposal based on a bevy of animal studies has not translated more convincingly into studies of neurophysiology and pathophysiology in humans. While it may yet achieve some therapeutic place, for now, we are left in an uncertain and problematic place. It is uncertain because there is always the possibility that subtle differences in experimental protocols and methods of measurement may obscure a genuine effect, and that to achieve a clinically useful effect (if it exists), the number of participants in studies may need to be increased many fold. It is problematic because early published clinical and other studies in humans pointed to a beneficial effect of even a single session of AIH on motor outcomes, but it is doubtful that CO_2_ levels were high enough to help promote the potential effect of AIH at motoneuronal level. As a result, we are unable to decide for or against the corollaries of the central dogma surrounding AIH.

Mindful that early studies in a field usually amplify the size of any real effect and that any *P*‐value of 0.05 has only a 50% chance of replication (Gandevia et al., 2021), we have therefore subjected the three papers (Christiansen et al. 2018; Finn et al. 2022; Welch et al., 2022) in this Connections piece to a formal Quality Output Checklist and Content Assessment (QuOCCA) (Héroux et al., 2022). The results are given in a Supplementary file S1. The findings of this independent assessment are likely typical of those in neuroscience and physiology, and they point to key areas for improvement which should enhance the reproducibility of the sorts of human studies discussed here.

## AUTHOR CONTRIBUTIONS

Both authors have read and approved the final version of this manuscript and agree to be accountable for all aspects of the work in ensuring that questions related to the accuracy or integrity of any part of the work are appropriately investigated and resolved. All persons designated as authors qualify for authorship, and all those who qualify for authorship are listed.

## CONFLICT OF INTEREST

None declared.

## Supporting information

Quality Output Checklist and Content Assessment

## Footnotes

1 Now published: Mathew, A.J., Finn, H.T., Carter, S.G., Gandevia, S.C. & Butler, J.E. (2024). Motor evoked potentials in the human upper and lower limb do not increase after single 30‐minute sessions of acute intermittent hypoxia. *Journal of Applied Physiology*, in press. 
