# Supplementary material for: Acute intermittent hypoxia: Enhancing motoneuronal output or not?
Source: Exp Physiol. 2024 Jun 25;109(9):1417–9. doi: 10.1113/EP091985 (PMC11363096; doi:10.1113/EP091985)
Supplement: Supplementary file 1 — Quality Output Checklist and Content Assessment [file EPH-109-1417-s001.docx]

|  | **QuOCCA** | **Christiansen et al. 2018** | **Finn et al. 2022** | **Welch et al. 2022** |
| --- | --- | --- | --- | --- |
| **Transparency** | **1a** Were the study’s hypotheses and analyses plan **registered** prior to the conduct of the study (i.e. pre-registered)? | No | No | No |
|  | **1b** If so, was the main conclusion reported in the abstract (or summary) based on the primary hypothesis/outcome? | NA | NA | NA |
|  | **2** Are the **primary data accessible** to independent researchers on a public website? | No | Yes | No |
|  | **3** Is **code used for the study available** on a public website to allow for reproduction or analysis of data? | No | No | No |
| **Design and Analysis** | **4** Was **ethics** approval obtained? | Yes | Yes | Yes |
|  | **5a** Was the **sample size** based on a formal sample size calculation done prior to starting the study? | No | Yes | No |
|  | **5b** If so, was the planned sample size adhered to? | NA | No | NA |
|  | **6** Was data analysis **blinded**? | No | Yes | No |
| **Reporting Practices** | **7** Are any **reporting guidelines** specified (such as those found at *www.equator-network.org*)? | No | No | No |
|  | **8a** Are all **measures of variability** defined in figures, tables and text? | Yes | Yes | No |
|  | **8b** Are any data summarised using standard error of the mean (SEM)? | No | No | No |
|  | **8c** If the SEM is used, are sample sizes specified for all reported SEM? | NA | NA | NA |
|  | **9a** Were any data excluded? | No | Yes | Yes |
|  | **9b** If so, was a criterion given? | NA | Yes | Yes |
|  | **10a** If null-hypothesis testing of significance was used, is a **probability threshold** specified for all statistical tests? | Yes | Yes | Yes |
|  | **10b** If used, are **exact probability values** used throughout the report, excluding figure legends? | No | No | No |
|  | **11** Are claims made for the importance or significance of results associated with a P-value greater than or equal to 0.05 (or other threshold) i.e. misleading spin of reported results? | No | No | No |

**Legend:** Assessment of the three ‘Connections’ articles, published in 2018 and 2022, using the Quality Output Checklist and Content Assessment (QuOCCA) (Héroux et al. 2022). This tool has 11 items that cover research transparency, research design and analysis, and research reporting practices. It is designed to help ensure that scientific work is open, reliable, and well-reported. Biomedical institutions and researchers can use it to evaluate publications, track changes in research practices, and encourage more transparent and rigorous science. An instructional guide on how to use the QuOCCA with detailed instructions and examples at <https://neura.edu.au/resources-tools/quocca>.
